# Increased plasma IL-17, IL-31, and IL-33 levels in chronic spontaneous urticaria

**DOI:** 10.1038/s41598-017-18187-z

**Published:** 2017-12-19

**Authors:** Wei Lin, Qiongyan Zhou, Chunbo Liu, Mengxia Ying, Suling Xu

**Affiliations:** 1 Department of Pharmacy, Affiliated Hospital of Ningbo University School of Medicine, Ningbo, 315020 China; 2 Department of Dermatology, Affiliated Hospital of Ningbo University School of Medicine, Ningbo, 315020 China; 3 Department of Nursing, Affiliated Hospital of Ningbo University School of Medicine, Ningbo, 315020 China

## Abstract

Chronic spontaneous urticaria (CSU) is considered in a subset of patients to be an autoimmune disorder. Interleukin(IL)-17, IL-31, and IL-33 are involved in some immune response. The aim of this study was to quantify plasma IL-17, IL-31, and IL-33 levels in CSU patients and to examine their relationships with disease severity. Plasma IL-17, IL-31, and IL-33 concentration were measured in 51 CSU patients and 20 healthy subjects (HCs). Plasma IL-17 (*P* < 0.001), IL-31 (*P* < 0.001), and IL-33 (*P* < 0.001) concentrations were significantly higher in CSU patients when compared with those of HCs. Concerning UAS7, severe group of CSU patients had significantly higher IL-17 levels than the moderate and mild groups (*P* = 0.028 and 0.007, respectively), and significantly higher IL-33 concentrations than the mild group (*P* = 0.026). Regarding only pruritus, severe group of patients had significantly higher IL-31 levels than the mild group (*P* = 0.003). The IL-33 levels in the total IgE positive group were significantly higher than that of negative group (*P* = 0.010). Our results showed higher plasma levels of IL-17, IL-31, and IL-33 among CSU patients which may highlight a functional role of these cytokines in the pathogenesis of CSU.

## Introduction

Chronic spontaneous urticaria (CSU) is a common and disabling disease characterized by recurrent itchy wheals and/or angioedema for more than 6 weeks due to known or unknown causes^1^. These symptoms are the consequence of skin mast cells degranulation with release of histamine and other vasoactive mediators. Autoantibodies (anti-IgE, anti-FcξRI) may be involved in only one-third of the cases^2^, suggesting that other circulating mediators, including cytokines, may be involved in the pathogenesis of CSU. The CSU shows both autoimmune and allergic disease characteristics^3^, which is associated with an imbalance between cytokines and T lymphocyte subgroups. Several data support the participation of interleukins in the pathophysiology of chronic urticaria^4,5^.

IL-17, IL-31, and IL-33 are multifunctional cytokines playing key roles in inflammation and immunity. IL-17 produced by CD4^+^T-helper subset that named T helper (Th) type 17^6^, is binding to an IL-17 receptor expressed on epithelial, endothelial, and fibroblastic stromal cells. IL-17 is associated with many autoimmune disorders, including rheumatoid arthritis, inflammatory bowel disease, multiple sclerosis and asthma^7,8^. IL-31 is mainly produced by Th2 cells^9^ and mast cells^10^. IL-31 acts on a broad range of immune- and non-immune cells and therefore possesses potential pleiotropic physiological functions, including regulating hematopoiesis and immune response, causing inflammatory bowel disease, airway hypersensitivity and dermatitis^9^. Moreover, IL-31 has also been described to play a key role in the pathogenesis of atopic dermatitis^11^ and contribute to itching via activation of the IL-31 receptor on sensory nerve cells^12^, therefore is considered to be an IL that can lead to skin inflammation. IL-33 is released in the extracellular space following cell injury. Its receptor ST2 is an IL-1R-related protein expressed on Th2 cells, mast cells, basophils and eosinophils^13,14^. Consequently, IL-33 has been shown to be involved in Th2-mediated immune responses, such as asthma, parasitic infections^15^, and atopic dermatitis^16^.

In these regard, IL-17, IL-31, and IL-33 might be involved in the pathogenesis of CSU, and their levels could be biomarker of disease severity or treatment response in CSU. So far, there are few available data regarding behavior of IL-17, 1L-31, and IL-33 in patients with CSU. Therefore, the aim of this study was to measure the values of plasma IL-17, 1L-31, and IL-33 levels in patients with CSU and analyze their relations to disease severity.

## Results

**Figure 1:**
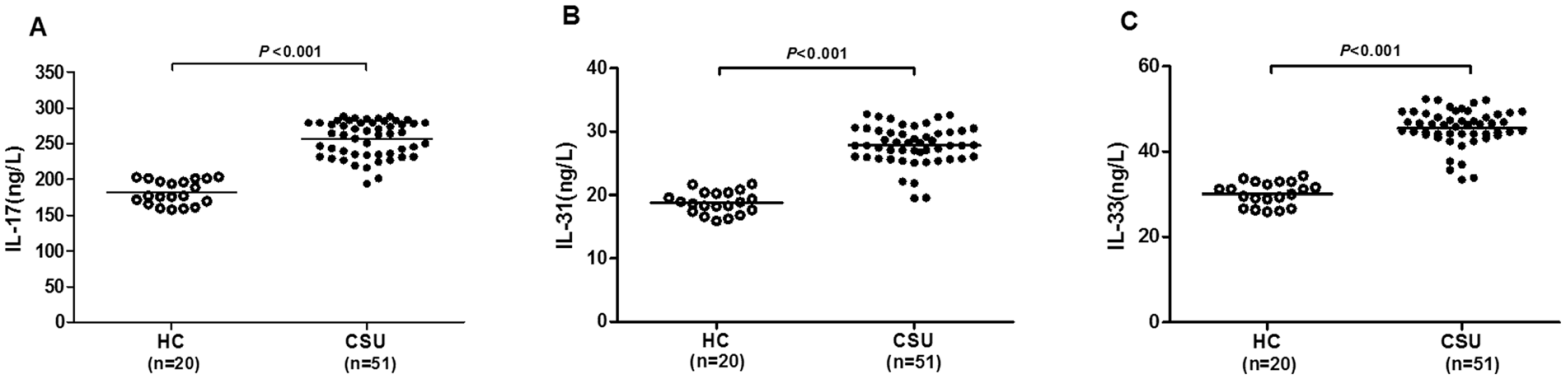
Comparison of IL-17, IL-31, and IL-33 levels in plasma between CSU patients and HCs. The levels of plasma IL-17 (*P* < 0.001) (**A**), IL-31 (*P* < 0.001) (**B**), and IL-33 (*P* < 0.001) (**C**) were significantly higher in CSU patients than those in HCs. Horizontal lines represent the mean values for IL-17, IL-31, and IL-33. CSU, chronic spontaneous urticaria; HC, healthy control.

Plasma levels of IL-17 (256.71 ± 25.07 ng/l *vs*. 181.79 ± 16.62 ng/l, *P* < 0.001; Fig. 1A), IL-31 (27.79 ± 3.02 ng/l *vs*. 18.78 ± 1.71 ng/l, *P* < 0.001; Fig. 1B), and IL-33(45.53 ± 4.32 ng/l *vs*. 30.09 ± 2.69 ng/l, *P* < 0.001; Fig. 1C) were significantly higher in CSU patients compared with those of healthy controls.

**Figure 2:**
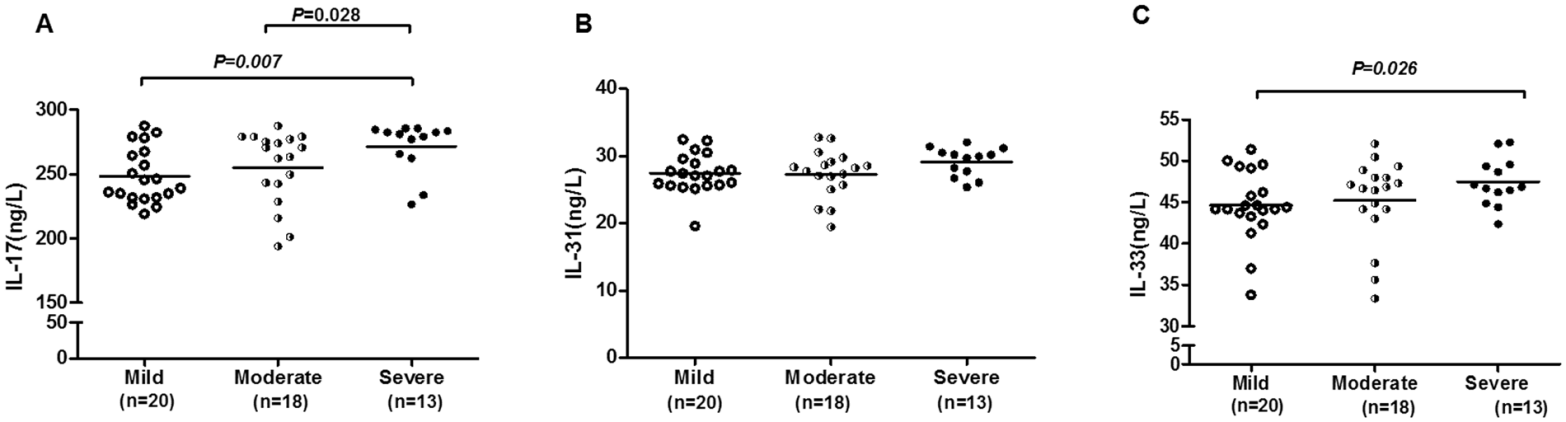
Comparison of IL-17, IL-31, and IL-33 levels according to disease severity in CSU patients. Severe group of CSU patients had significantly higher IL-17 levels than the moderate and mild groups (*P* = 0.028 and 0.007, respectively) (**A**); no significant differences were found in IL-31 levels between different severity group (**B**). An increased level of IL-33 was observed in severe group of CSU patients compared with the mild group (*P* = 0.026), no significant differences in IL-33 levels between mild and moderate group or moderate and severe group (**C**).

**Figure 3:**
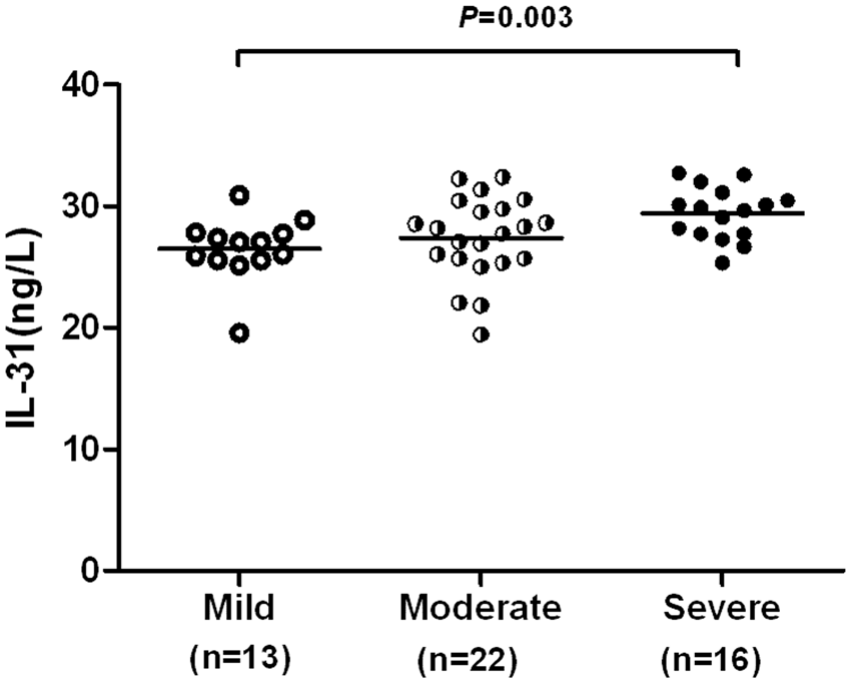
Comparison of IL-31 levels according to pruritus intensity in CSU patients. An increased level of IL-31 was observed in severe group of CSU patients compared with the mild group (*P* = 0.003), but no significant differences between mild and moderate group or moderate and severe group.

Concerning the urticaria activity, severe group (271.51 ± 19.76 ng/l) of CSU patients had significantly higher IL-17 levels than the moderate (255.21 ± 28.56 ng/l) and mild (248.44 ± 21.30 ng/l) groups (*P* = 0.028 and 0.007, respectively; Fig. 2A). There was no significant differences in IL-31 levels between different severity group (Fig. 2B). An increased level of IL-33 was observed in severe group (47.41 ± 2.88 ng/l) of CSU patients compared with the mild group (44.62 ± 4.24 ng/l, *P* = 0.026; Fig. 2C), but no significant differences in IL-33 levels between mild and moderate (45.19 ± 5.04 ng/l) group or moderate and severe group. However, regarding only pruritus, severe (29.41 ± 2.15 ng/l) group of CSU patients had significantly higher IL-31 levels than the mild group (26.49 ± 2.62 ng/l, *P* = 0.003; Fig. 3), no significant differences between mild and moderate (27.39 ± 3.38 ng/l) group or moderate and severe group.

**Figure 4:**
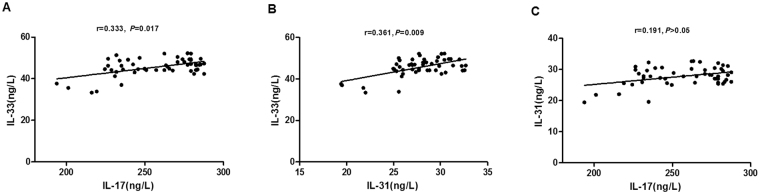
Correlation of IL-17, IL-31, and IL-33 levels in plasma. Both IL-17 (r = 0.333, *P* = 0.017) (**A**) and IL-31 (r = 0.361, *P* = 0.009) (**B**) were significantly correlated with IL-33 levels; no significant correlation was found between IL-17 and IL-31 (r = 0.191, *P* > 0.05) (**C**).

We evaluated the correlation of IL-17, IL-31, and IL-33 in plasma by the Spearman’s rank test. Interestingly, both IL-17 (r = 0.333, *P* = 0.017; Fig. 4A) and IL-31 (r = 0.361, *P* = 0.009; Fig. 4B) were significantly correlated with IL-33 levels. Nevertheless, the levels of IL-17 in plasma were not relevant to that of IL-31 (Fig. 4C).

**Figure 5:**
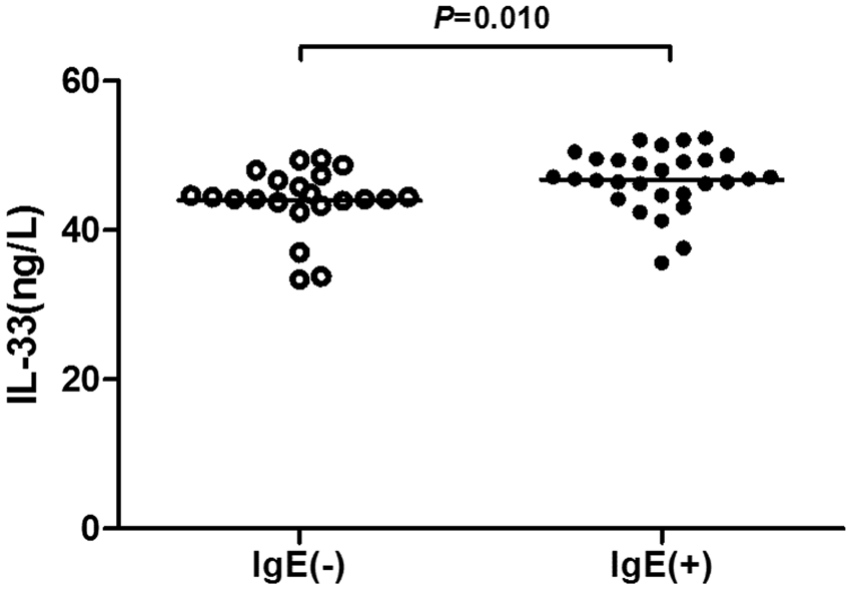
Comparison of IL-33 between IgE positive group and negative group. The level of IL-33 was significantly higher in the total IgE positive group than that of negative group (*P* = 0.010).

A higher level of IL-33 was observed in the total IgE positive group compared with that of negative group (46.73 ± 4.02 *vs* 43.96 ± 4.31 ng/l, *P* = 0.010; Fig. 5), but not for IL-17 and IL-31. There were no significant differences in IL-17, IL-31, or IL-33 levels according to the age, gender and presence of angioedema. Plasma levels of IL-17, IL-31, and IL-33 were not significantly correlated with CRP or blood eosinophil count.

## Discussion

A full understanding of the pathogenesis of CSU has yet to be achieved. In the present study, to gain better understanding of the role of cytokines in immunopathogenesis of CSU we aimed to determine whether CSU is associated with alterations in IL-17, IL-31, and IL-33. The results of our study showed that plasma levels of IL-17, IL-31, and IL-33 were significantly elevated in patients with CSU. Severe group of CSU patients had significantly higher IL-17 levels compared with the moderate and mild group, and significantly higher IL-33 concentration than the mild group of CSU patients.

The IL-17 levels were significantly higher in CSU patients compared with the control group, and severe group had significantly higher IL-17 levels compared with the other two groups of CSU patients. In agreement with our results, many studies showed that the IL-17 levels of CU patients was higher than that of control^17–19^, and there were significant positive correlation between serum IL-17, IL-23, TNF-α and disease activity^18^. In another report, IL-17 were significantly higher in autologous serum skin test (ASST) positivity than in ASST negative CSU patients^20^. Moreover, patients with CSU and ASST positivity, showed increased circulating levels of TNF-α, IL-1β and IL-6. These pro-inflammatory cytokines, in turn, are known to be induced by IL-17, which may contribute to the inflammatory profile founded in CSU^17^.

The important role of IL-31 in atopic dermatitis, in particular its impact on intensity of pruritus, is well known. An enhanced expression of the specific IL-31RA was discovered in cells of the human and murine dorsal root ganglia and in murine primary afferent fibers of the spinal cord and dermis that are proposed to be involved in the sensation of itch^21,22^. Furthermore, IL-31 antibodies have been shown to reduce itch significantly in a mouse model of AD^23^, confirming in patients with moderate-to-severe AD very recently^24^. Our results agreed with a previous study, in which the researchers demonstrated that IL-31 levels in CSU patients were significantly higher compared with those of controls^25^. However, there was no difference in IL-31 plasma levels in ASST positive or negative CU patients^25^. We could not find a correlation between IL-31 plasma levels and the urticaria activity, confirming a previous report^26^, but if regarding only pruritus, severe group of CSU patients had significantly higher IL-31 levels than the mild group. This may be attributed to the fact that IL-31 is contribute to itching.

IL-33 is being increasingly recognized as an important inflammatory cytokine. The plasma levels of IL-33 were significantly higher in patients with CSU, and severe group had significantly higher concentration compared to the mild group of CSU patients. In support of our finding, elevation of IL-33 was recently demonstrated in the lesional skin of CSU patients^27^. Besides, among the patients who had received desloratadine for two weeks, there was a significant reduction in IL33 levels of CSU patients^28^. IL-33 induces increased release of Th2 cytokines such as IL-5 and IL-13 from Th2 cells *in vitro* and elevated levels of plasma IgE and blood eosinophils *in vivo* ^29^. IL-33 also causes activation, maturation, and Th2 cytokine production in mast cells^30^ and induces eosinophil-dependent cutaneous fibrosis^27^. Thus, IL-33 may play a pivotal role in the development of inflammatory reactions in CSU.

Inconsistent with our results, there were studies demonstrated the plasma IL-17 levels in CSU patients were not differ from the healthy control^20,31^ or even lower^32^. Besides, two reports showed that there was no significant difference in plasma IL-33 levels between patients with urticaria and control subjects^33,34^. The probable cause of the different result may be impact of genetic variation in the study population or the study was conducted on a small number of patients^32^.

Intriguingly, IL-33 levels were both correlated with IL-17 and IL-31 in CSU patients. Many studies provided indirect evidence for a functional link between these cytokines in many human diseases. Vocca *et al*. reported IL-33/ST2 axis was involved in Th2/IL-31 and Th17 immune response during the progression of allergic airway disease^35^. Nygaard *et al*. found a moderate, positive correlation between IL-33 and IL-31 in atopic dermatitis^36^. These authors speculate that the activation of the IL-33-ST2 axis, as a biomarker of Th2/IL-31 immune response, may be a critical crossroad between the immune system and epidermal homoeostasis^36^. On the other hand, Meephansan *et al*. found IL-17A induced IL-33 in epidermis through EGFR, EPK, p38 and JAK/STAT1 pathways, which were necessary for induction of IL-33^37^. There may be a functional link between these cytokines, but the exact mechanism is not yet clear and needs further study.

A correlation between IL-33 and IgE has been reported. A study demonstrated that mast cells produce IL-33 after IgE-mediated activation and that the IL-33/ST2 pathway was critical for the progression of IgE-dependent inflammation^38^. Futhermore, IL-33 enhances IgE-mediated degranulation and migration as well as IgE- and IL-3-mediated cytokine and chemokine production in human and mouse basophils^39^. On the other hand, long-term exposure of human and mouse muscle cell to IL-33 resulted in attenuation of IgE/Ag-FcεRI-mediated degranulation due to down-regulation of PLCγ1 and Hck expression, although short term exposure to IL-33 did not influence that degranulation directly^40^. In our study, we found the IL-33 levels in the total IgE positive group were significantly higher than that of negative group, providing evidence for this functional link between IL-33 and IgE in CSU.

Our study has two limitations. One is the relatively small number of study subjects. The other is the absence of a positive control group, such as atopic dermatitis or psoriasis as an inflammatory dermatosis. Therefore, further studies with a larger sample size and a positive control group are required to confirm our results.

In summary, our results showed high plasma levels of IL-17, IL-31, and IL-33 among CSU patients which may highlight a functional role of these cytokines in the pathogenesis of this common skin disease, and may provide the rationale for new treatment strategies in chronic urticaria. However, more studies are needed on more patients to study different Th1, Th2 and Th17 cytokines in plasma and skin of CSU patients.

## Methods

### Study subjects

We studied 51 CSU patients and 20 sex-matched and age-matched healthy controls as control. CUS was diagnosed according to the EAACI/GA^2−^LEN/EDF/WAO guidelines. We excluded patients with clinical evidence of urticaria vasculitis and physical urticaria, such as dermographism, cholinergic urticaria, and cold urticaria. Anti-histamines were discontinued 1 week before the study and none of the patients was taking any other drugs for more than 8 weeks preceding the study. All the controls did not take any medication for at least 2 weeks before the study. Disease activity in all CSU patients was determined by use of UAS7 during 7 days. Weekly UAS were graded as follows: 0–14(mild), 15–28(moderate) and 29–42(severe). Weekly pruritus intensity was graded as: 0–7(mild), 8–14(moderate) and 15–21(severe). The characteristics of patients (n = 51) are shown in Table 1.

**Table 1:** Clinical characteristics of patients with CSU and HC.

Variable	CSU(n = 51)	HC(n = 20)	*P*-value
Age (year)	28 ± 13	32 ± 14	0.285
<20	12/51 (23.5%)		
20–30	22/51 (43.1%)		
>30	17/51 (33.3%)		
Gender (male)	16/51 (31.4%)	6/20 (30.0%)	0.91
Disease duration (month)	12.3 ± 20.2		
Presence of angioedema	17/50 (34.0%)		
Blood eosinophils (×10^6^/L)	115.5 ± 106.4		
C- reactive protein	3.8 ± 13.0		
Total IgE positive	29 (56.9%)		
Disease severity			
Mild	20/51 (39.2%)		
Moderate	18/51 (35.3%)		
Severe	13/51 (25.4%)		

Total IgE were measured using immunoblot assay (MEDIWISS Analytic GmbH, Moers, Germany), when the value higher than 100 kU/l meant positive.

The study protocol was approved by the Institutional Review Board for Human Studies of Affiliated Hospital, School of Medicine, Ningbo University (Ningbo, China). All subjects provided written informed consent before participation and methods in this study were performed in accordance with the relevant guidelines and regulations.

### Assay of IL-17, IL-31, and IL-33

Plasma was collected and stored at −80 °C. Concentration of IL-17, IL-31, and IL-33 in plasma was measured by the enzyme-linked immunosorbent assay (ELISA) using a commercial kit (IL-17: Shanghai Future Industry Co., Ltd., Shanghai, China; IL-31: Shanghai Future Industry Co., Ltd., Shanghai, China; IL-33: Shanghai Future Industry Co., Ltd., Shanghai, China;). The assays were conducted according to the manufacturer’s guidelines. The samples were analyzed in batches to minimize interassay variability.

### Statistical analysis

Data were delivered as medians and ranges. Kruskal-Wallis variance analysis was used for screening differences between the groups. Mann–Whitney *U* test was used to compare data between the patient groups and the healthy controls. Spearman’s rank test was used for correlations. The probability value of *P* < 0.05 was assumed significant. The data were analyzed with SPSS statistics 18.0.
